# The ‘July Effect’ in supervisory residents: assessing the emotions of rising internal medicine PGY2 residents and the impact of an orientation retreat

**DOI:** 10.1080/10872981.2020.1728168

**Published:** 2020-03-09

**Authors:** Garth W. Strohbehn, Kathryn Levy, Phoebe A. Tsao, Daniel T Cronin, Lauren A. Heidemann, John Del Valle

**Affiliations:** a Internal Medicine Residency Program, Department of Internal Medicine, University of Michigan Medical School, Ann Arbor, MI, USA; b Division of Hospital Medicine, Department of Internal Medicine, University of Michigan Medical School, Ann Arbor, MI, USA

**Keywords:** July Effect, graduate medical education, mentorship

## Abstract

**Background**: The arrival of new residents brings challenges for residency programs and residents. Many residency programs conduct orientation sessions to help transition rising supervisory residents into their new roles, but no evaluation of their impact on residents’ emotional well-being has been performed.

**Objective**: This study assesses the impact of a half-day orientation retreat on rising internal medicine post-graduate year (PGY) 2 residents’ emotions toward PGY2 year and their self-confidence in fulfilling the supervisory resident role.

**Design**: A survey was administered to a class of rising supervisory residents immediately before and after an orientation retreat in May 2017. The survey provided participants an open-ended prompt to describe their emotions toward PGY2 year and a 5-point Likert scale to rate their confidence in fulfilling supervisory resident roles. Differences were assessed using McNemar’s exact and Wilcoxon signed-rank tests, respectively.

**Results**: Forty-four of 50 (88%) eligible participants completed pre- and post-intervention Likert scales and 40 of 50 (80%) eligible participants completed corresponding emotion sections. Pre-intervention the most common emotions were anxiety (n = 33, 82.5%) and excitement (n = 32, 80.0%). Post-intervention, participants’ fear was reduced (45.0% vs 12.0%; p < 0.001). Participants reported greater confidence that internship prepared them for PGY2 year and understanding of triaging and admitting principles (agree or strongly agree from 65.9% to 84.0% and from 25.0% to 68.2%, respectively; p < 0.005 for improvement by Wilcoxon signed-rank for both).

**Conclusions**: Orientation retreats may be an effective way to reduce fear and demystify the supervisory resident role.

## Introduction

The existence of a deleterious effect on patient care that accompanies the annual cohort turnover of residents – colloquially referred to as the ‘July Effect’ – continues to be debated in the literature [1–6]. In practice, however, many internal medicine residency program leaders believe in the July Effect’s existence and have invested resources in combatting it [7]. While most of the interventions are didactic and target new post-graduate year (PGY) 1 residents, [7,8] approximately 60% of programs conduct orientation programs to support rising PGY2 residents transitioning into supervisory roles [7,9–15].

Since Wipf and colleagues first published about their transition course for their new PGY2 medicine residents at the University of Washington in 1995 [10], other institutions have replicated or adapted their work to create courses for their rising senior residents [7,9–13]. In line with prior needs assessments, these orientations typically focus on leadership and teaching skills [9,11,13,14]. Anecdotal evidence hints at the efficacy of rising PGY2 retreats [15] and limited quantitative assessment suggests an improvement in participants’ team-building capacity [9], but critically, these retreats have not to date been evaluated through the lens of resident wellness or emotional well-being.

Similar to peer internal medicine residency programs, our program has implemented a half-day orientation retreat to orient rising supervisory residents to their upcoming roles. In this study, we aimed to analyze its effect on their emotions and self-assessed preparedness for the supervisory medical resident’s roles. The over-arching goal of the project was to begin to apply measurement and statistical evaluation to educational curricula and wellness to better inform future iterations of the curricula.

## Methods

### Setting

The study was designed by University of Michigan internal medicine chief residents (CMR). Rising PGY2 internal medicine and internal medicine-pediatric residents attended a CMR-led half-day retreat in May 2017 in a non-clinical environment. Local Institutional Review Board (IRB) deemed the study exempt (HUM00130363).

### Participants

Participants included rising PGY2 residents from our internal medicine and internal medicine-pediatrics program. Our program collectively refers to all internal medicine and internal medicine-pediatric residents beyond PGY1 year as ‘senior medical residents’. These senior medical residents oversee the clinical activities of PGY1 residents and medical students, provide education and feedback to those PGY1 residents and medical students, work with hospitalists, subspecialist, and Emergency Department physicians to triage patients, and serve as the primary point of contact for the inpatient attending physician of record.

### Survey instrument

An anonymous paper survey instrument was administered before and after the intervention. Pre- and post-intervention surveys were anonymous and matched for paired analysis by survey number. The survey contained two sections. First, it asked participants to rate on a 5-point Likert scale their level of agreement with a series of statements about the roles of supervisory medical residents with focus on their confidence in fulfilling certain tasks (1 = Strongly disagree, 2 = Disagree, 3 = Neutral, 4 = Agree, 5 = Strongly agree). Second, it invited participants to provide up to five single-word descriptions of their emotions toward being a supervisory medical resident. To assess for systematic over-rating following the intervention (i.e., Hawthorne effect), we included a ‘dummy’ question pertaining to a topic that was not specifically addressed in the intervention: *‘I am able to recognize “sick” from “not sick”’* These sections were repeated in the post-intervention survey. The survey instruments are available in Supplementary Appendix A.

### Interventions

Our orientation to senior year retreat was divided into lecture and small group breakout sections [10]. Lectures were divided into subsections detailing the four major roles of the PGY2 resident: Clinician, Community Member and Trendsetter, Teacher and Adult Learner, and Manager. Guest faculty speakers and CMRs each provided approximately one-half of lecture-based content. Select lecture-based orientation materials are included in Supplementary Appendix B. Current senior medical residents, pre-selected and invited by CMRs, conducted the one-hour small group breakout sessions with a roundtable, question-and-answer format.

### Outcome analysis

We performed all statistical analysis in Microsoft Excel (Redmond, WA). We generated word clouds of emotions using a freely available online applet (https://www.Jasondavies.com/wordcloud; accessed February 2018). Two authors (KL, PAT) independently coded each emotion into one of the 27 categories as defined by Cowen & Keltner [16]. Emotions that did not clearly fall into pre-determined categories were sorted according to raters’ best judgment, and any categorization discrepancies were discussed between the two raters until a mutual agreement was reached.

We calculated differences in participants’ level of agreement with the series of statements pertaining to the roles of supervisory medical residents and assessed for its statistical significance using Wilcoxon signed-rank test with post hoc Bonferroni correction. We calculated the differences in likelihood of an individual experiencing a given emotion and assessed for its statistical significance using McNemar’s exact test for paired nominal data (no post-hoc correction). For all tests of significance, we generally expected intervention to *improve* experiences. Therefore, one-sided alpha of 0.05 was used as a critical value for statistical significance.

## Results

### Analysis of emotions and effect of the intervention on emotions

**Figure 1.**
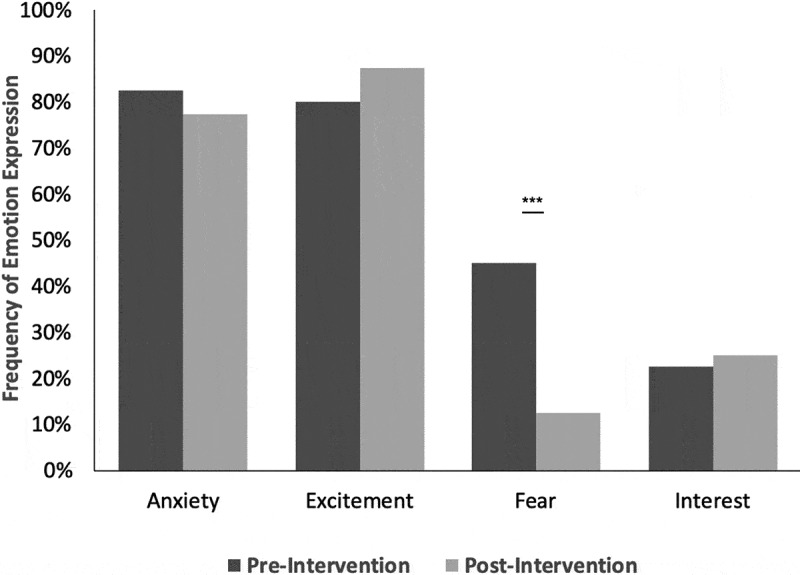
Effect of orientation retreat on rising PGY2 emotions. Emotion frequencies prior to (dark bars) and following (light bars) orientation retreat. Statistical significance by McNemar’s exact test indicated by ‘***’ for p < 0.001

Forty-four of 50 eligible residents completed both pre- and post-intervention surveys (response rate, 88%). Word clouds of reported emotions were generated from pre-intervention (Supplementary Figure 1a) and post-intervention (Supplementary Figure 1b) survey results. Emotions clustered into four predominant categories: Anxiety, excitement, fear, and interest (Table 1). Initial inter-rater agreement for categorization of listed emotions was 79.4% (204 of 257) overall and 85.1% (189 of 222) for high-frequency categories of emotions. Fear toward PGY2 year was significantly lower post-intervention (45.0% vs 12.0%; p < 0.001) (Figure 1; Table 1 row 3). Anxiety, excitement, and interest did not significantly change following the intervention.

**Table 1.** Frequencies of emotions experienced by rising PGY2 residents toward the supervisory resident role

Emotion category^a^	Pre-intervention number of participants experiencing (% of participants experiencing)	Post-intervention number of participants experiencing (% of participants experiencing)
Anxiety	33 (82.5)	31 (77.5)
Excitement	32 (80.0)	35 (87.5)
Fear***	18 (45.0)	5 (12.5)
Interest	9 (22.5)	10 (25.0)
Admiration	6 (15.0)	3 (7.5)
Calmness	4 (10.0)	4 (10.0)
Joy	3 (7.5)	1 (2.5)
Relief	3 (7.5)	3 (7.5)
Confusion	2 (5.0)	0 (0.0)
Horror	2 (5.0)	0 (0.0)
Boredom	1 (2.5)	1 (2.5)
Surprise	0 (0.0)	1 (2.5)

^a^Remainder of categories had n = 0 for both pre- and post-intervention: adoration, aesthetic appreciation, amusement, anger, awe, awkwardness, craving, disgust, emphatic pain, entrancement, nostalgia, romance, sadness, satisfaction, and sexual desire. ‘***’ indicates p < 0.005 by McNemar exact test. Expression of each category of emotion (column 1) prior to (column 2) and following (column 3) orientation retreat. ^a^Remainder of categories had n = 0 for both pre- and post-intervention: adoration, aesthetic appreciation, amusement, anger, awe, awkwardness, craving, disgust, emphatic pain, entrancement, nostalgia, romance, sadness, satisfaction, and sexual desire. Statistical significance by McNemar’s exact test indicated by ‘***’ for p < 0.001.

### Resident comfort with roles and responsibilities

**Figure 2.**
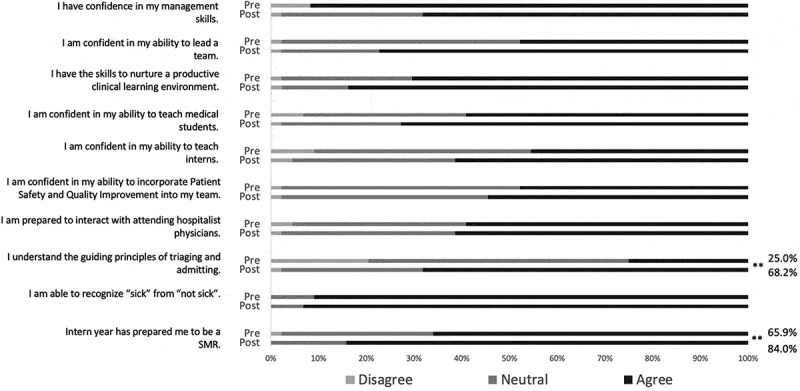
Improvement in rising PGY2 confidence toward the supervisory resident role after orientation retreat. Quantitative analysis of level of agreement with statements in the Likert scale survey. Statistical significance by Wilcoxon signed-rank test indicated by ‘**’ for p < 0.005. Percentage of participants responding ‘agree’ or ‘strongly agree’ is included for statistically significant results

Pre-intervention and post-intervention results of the Likert survey are summarized in Figure 2. Participants gained comfort in the general principles of triaging and admitting, from 25.0% comfort pre-intervention to 68.2% comfort post-intervention. (p < 0.005) Confidence that PGY1 year had prepared participants for the supervisory resident role increased, as well, from 65.9% confidence pre-intervention to 84.0% confidence post-intervention (p < 0.05). There was no significant difference in participants’ pre-intervention and post-intervention level of agreement with the statement *‘I am able to recognize “sick” from “not sick”’*, suggesting participants’ attentiveness to the survey.

## Discussion

This study provides both qualitative and quantitative assessment of a common curricular intervention for rising PGY2 internal medicine residents at a single residency program. It demonstrates the effectiveness of an orientation in reducing fear toward the supervisory resident role, improving self-assessed comfort with the guiding principles of triaging and admitting, and raising confidence that internship had prepared these residents for their coming responsibilities. These data help to confirm prior anecdotal evidence [15] and, importantly, serve as a starting point for further educational quality improvement work. Quantification of curricular interventions allows for year-on-year content updates to be measured and can help guide multiple plan-do-study-act cycles. Additionally, directly assessing emotions and their responses to interventions may be an under-recognized and important outcome measure from the standpoint of resident wellness.

Strengths of the study include a high survey response rate, the quantitative evaluation of multiple facets of the transition from PGY1 to PGY2, the simplicity of its design, and the unobtrusive nature of outcome assessment. However, the study is not without limitations. First, it is a single-center study at a tertiary-care institution and therefore may not be generalizable to other academic or community residency programs. Second, the outcome measures of choice are self-reported and therefore focus exclusively on the resident’s perceived comfort. Finally, this study represents a single data point in time. Alternatives in the future could include designing and validating a more clinically relevant outcome measure, multi-year evaluations, more formal testing of knowledge of service triaging, and re-assessment of the relevant outcome measures immediately at the beginning of PGY2 year.

In conclusion, in this study, we utilized a simple study design and unobtrusive survey to identify and quantify key emotions experienced by rising supervisory residents, most notably fear, and we demonstrate that an orientation retreat targeting these rising supervisory residents can reduce that fear. Moreover, we demonstrate that didactic lectures can improve self-assessed preparedness for the role. This work serves as a starting point for further investigation and quality improvement of medical education curricula.

## Supplementary Material

Supplemental Material
