# Supplementary material for: The ‘July Effect’ in supervisory residents: assessing the emotions of rising internal medicine PGY2 residents and the impact of an orientation retreat
Source: Med Educ Online. 2020 Mar 9;25(1):1728168. doi: 10.1080/10872981.2020.1728168 (PMC7144188; doi:10.1080/10872981.2020.1728168)
Supplement: Supplemental Material [file ZMEO_A_1728168_SM3669.zip › Supplementary/Strohbehn_et_al_Final_supplemental_methods_appendices_clean.docx]

**Supplemental Methods Appendix for:**

*The “July Effect” in supervisory residents: Assessing the emotions of rising internal medicine PGY2 residents and the impact of an orientation retreat*

**Authors:** Garth W Strohbehn, MD, MPhil^1*^; Kathryn Levy, MD^1,2^; Phoebe A Tsao, MD^1*^; Daniel T Cronin, MD^1,2^; Lauren A Heidemann, MD^1,2^; and John Del Valle, MD^1^

**Affiliations:** Internal Medicine Residency Program (1) and Division of Hospital Medicine (2), Department of Internal Medicine, University of Michigan Medical School, Ann Arbor, MI, USA, 48109

**Institutional Roles of Authors:** Chief Medical Residents (GWS, KL, DTC, PAT); Assistant Professor (LAH); Senior Associate Chair for Graduate Medical Education, Residency Program Director, and Professor of Gastroenterology (JDV)

***Current Affiliations:** Section of Hematology/Oncology, University of Chicago Medicine, Chicago, IL (GWS); Division of Hematology/Oncology, University of Michigan Medical School (PAT)

**Appendix A – Survey Instruments**

NB regarding formatting: On the original survey instruments the institutional review board approval number was included as a footer rather than within the body of the document.

**Intern Retreat**

**Pre-Retreat Survey**

Survey Number: __________

|  | Strongly Disagree | Disagree | Neutral | Agree | Strongly Agree |
| --- | --- | --- | --- | --- | --- |
| Intern year has prepared me to be a SMR. |  |  |  |  |  |
| I am able to recognize “sick” from “not sick”. |  |  |  |  |  |
| I understand the guiding principles of triaging and admitting. |  |  |  |  |  |
| I am prepared to interact with attending hospitalist physicians. |  |  |  |  |  |
| I will incorporate Patient Safety and Quality Improvement into my team. |  |  |  |  |  |
| I am confident in my ability to teach interns. |  |  |  |  |  |
| I am confident in my ability to teach medical students. |  |  |  |  |  |
| I have the skills to nurture a productive clinical learning environment. |  |  |  |  |  |
| I am confident in my ability to lead a team. |  |  |  |  |  |
| I have confidence in my management skills. |  |  |  |  |  |

1. Please list up to 5 words (single words only) describing your emotions toward being a SMR.

1.

2.

3.

4.

5.

2. Today I would like to learn about:

-

-

-

-

-

HUM00130363

**Intern Retreat**

**Post-Retreat Survey**

Survey Number: __________

|  | Strongly Disagree | Disagree | Neutral | Agree | Strongly Agree |
| --- | --- | --- | --- | --- | --- |
| Intern year has prepared me to be a SMR. |  |  |  |  |  |
| I am able to recognize “sick” from “not sick”. |  |  |  |  |  |
| I understand the guiding principles of triaging and admitting. |  |  |  |  |  |
| I am prepared to interact with attending hospitalist physicians. |  |  |  |  |  |
| I will incorporate Patient Safety and Quality Improvement into my team. |  |  |  |  |  |
| I am confident in my ability to teach interns. |  |  |  |  |  |
| I am confident in my ability to teach medical students. |  |  |  |  |  |
| I have the skills to nurture a productive clinical learning environment. |  |  |  |  |  |
| I am confident in my ability to lead a team. |  |  |  |  |  |
| I have confidence in my management skills. |  |  |  |  |  |

1. Please list up to 5 words (single words only) describing your emotions toward being a SMR.

1.

2.

3.

4.

5.

**COMMENTS:**

- Please provide any suggestions that will help us improve the retreat for next year. Also please comment on things you liked today and things you didn’t.
- Advice for the incoming interns: (we’ll share this with them at their orientation)

*If I only knew this when I started* … (doesn’t have to be about medicine)

HUM00130363

**Appendix B – Sample Orientation Content**


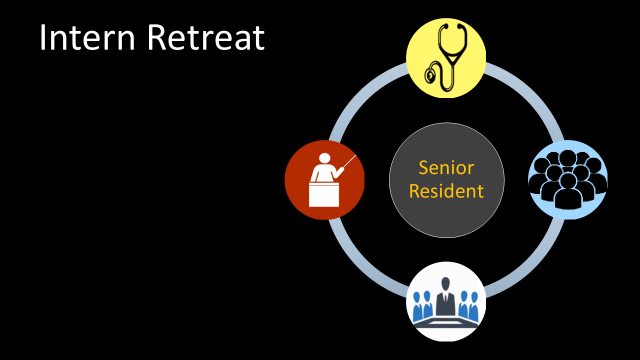


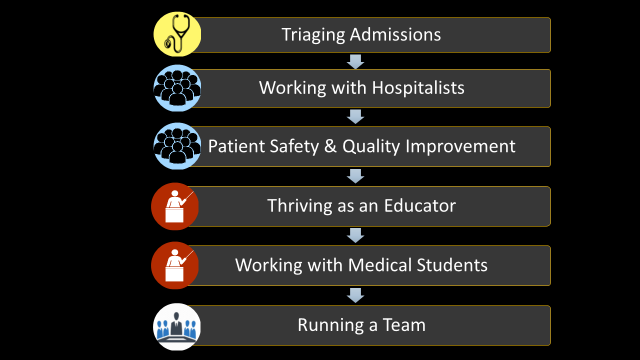


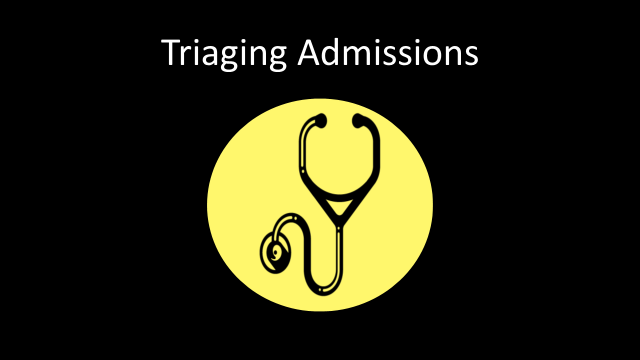


**
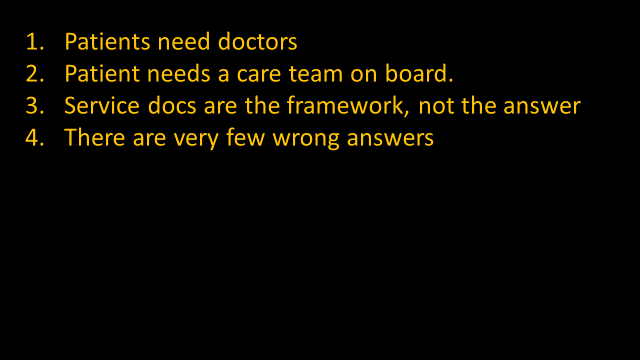
**

**
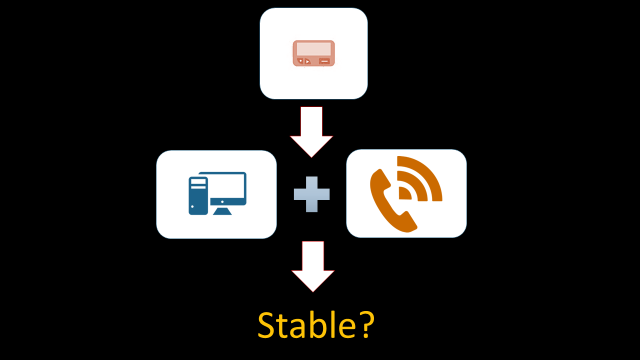
**

**
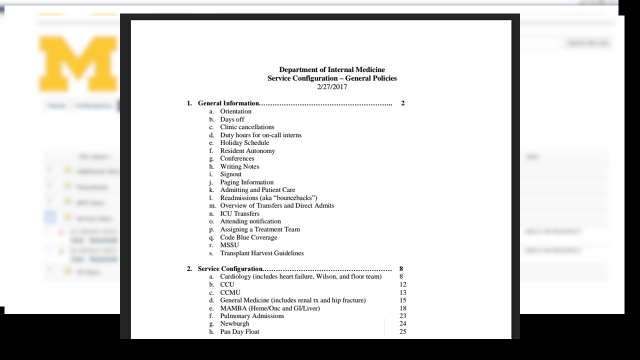
**

**
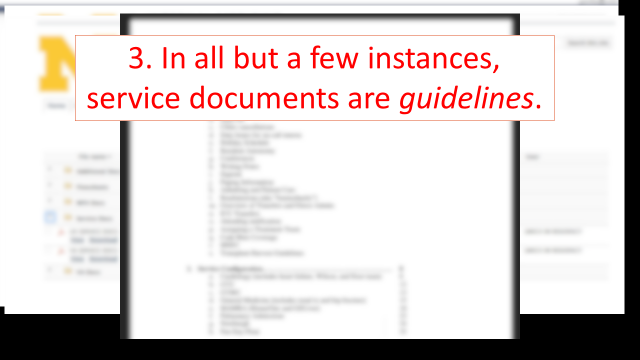
**

**
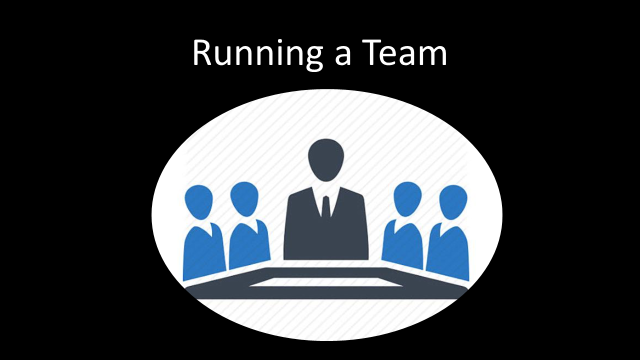
**

**
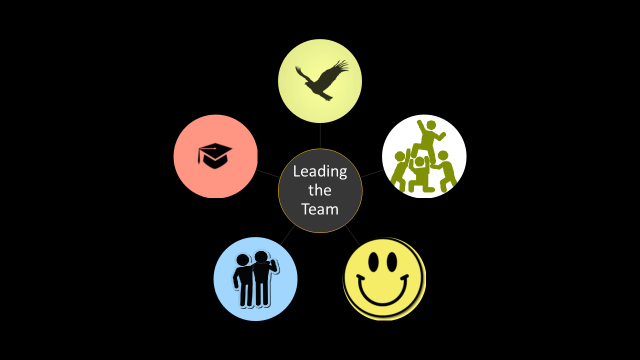
**
