# Supplementary material for: The ‘July Effect’ in supervisory residents: assessing the emotions of rising internal medicine PGY2 residents and the impact of an orientation retreat
Source: Med Educ Online. 2020 Mar 9;25(1):1728168. doi: 10.1080/10872981.2020.1728168 (PMC7144188; doi:10.1080/10872981.2020.1728168)
Supplement: Supplemental Material [file ZMEO_A_1728168_SM3669.zip › Supplementary/Supplememtary figure caption.docx]

Supplemental Figure 1. Word cloud of rising PGY2s’ emotions toward the supervisory resident role. (A) Word cloud of resident emotions prior to intervention. (B) Word cloud of resident emotions following intervention.
