# Supplementary figures and images for: The ‘July Effect’ in supervisory residents: assessing the emotions of rising internal medicine PGY2 residents and the impact of an orientation retreat
Source: Med Educ Online. 2020 Mar 9;25(1):1728168. doi: 10.1080/10872981.2020.1728168 (PMC7144188; doi:10.1080/10872981.2020.1728168)

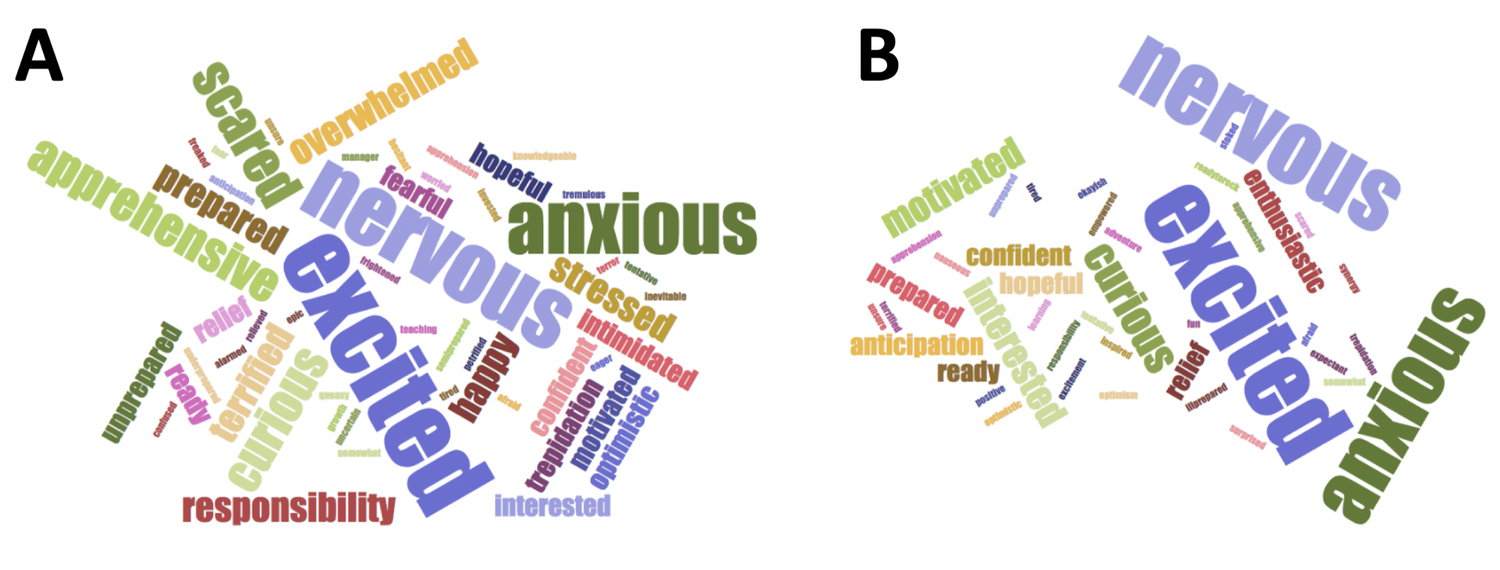

Supplement: Supplemental Material [file ZMEO_A_1728168_SM3669.zip › Supplementary/Supplemental_Figure_1.tif]
